# Cystic Fibrosis and Colorectal Cancer Risk: Reprogramming of the Intestinal Epithelial Niche and Cell‐State Plasticity in the CFTR Modulator Era

**DOI:** 10.1111/cpr.70272

**Published:** 2026-08-16

**Authors:** Bala Umashankar, Ines Pankonien, Margarida Amaral, Chee Y. Ooi, Thierry Jardé, Shafagh A. Waters

**Affiliations:** ^1^ School of Biomedical Sciences, Faculty of Medicine and Health UNSW Sydney Sydney New South Wales Australia; ^2^ Molecular and Integrative Cystic Fibrosis (miCF) Research Centre UNSW Sydney Sydney New South Wales Australia; ^3^ BioISI—Biosystems & Integrative Sciences Institute, Faculty of Sciences University of Lisbon Lisbon Portugal; ^4^ Department of Gastroenterology Sydney Children's Hospitals Network Sydney New South Wales Australia; ^5^ Sydney Children's Hospital Respiratory Department Sydney Children's Hospitals Network Sydney New South Wales Australia; ^6^ Department of Anatomy and Developmental Biology Monash University Clayton Victoria Australia; ^7^ Monash Biomedicine Discovery Institute Clayton Victoria Australia

**Keywords:** CFTR, CFTR modulator therapy, colorectal cancer, cystic fibrosis, epithelial–mesenchymal plasticity, intestinal stem cells

## Abstract

Cystic fibrosis (CF) has shifted from a fatal paediatric lung disease to a multi‐organ, lifespan‐spanning disorder in which gastrointestinal (GI) complications and malignancies are increasingly prominent. With improved survival into mid‐ and late adulthood, aided by newborn screening, optimised nutrition and the advent of highly effective CF transmembrane conductance regulator (CFTR) modulators, implementation of colonoscopic surveillance has highlighted a several‐fold increase in early‐onset colorectal cancer (CRC) and a broader spectrum of intestinal pathology. In parallel, work in mouse models, human tissue and patient‐derived intestinal organoids now places the CFTR at the centre of a complex epithelial compartment that integrates ion and pH homeostasis, mucus biology, microbiota, redox balance and immune/stromal function. In this review, we advance the hypothesis that CF may be conceptualised as a niche‐centric hereditary CRC predisposition syndrome, in which germline CFTR dysfunction chronically destabilises epithelial identity in addition to increasing mutational burden. We synthesise evidence that CFTR loss remodels stem cell regulation, promotes hypoxia and oxidative stress, perturbs microbial ecosystems, drives chronic immune activation and stromal remodelling, and induces epithelial–mesenchymal plasticity (EMP) and DNA‐damage vulnerability, collectively creating a pre‐neoplastic intestinal ecosystem. We situate this model within contemporary CRC frameworks that emphasise cell‐state transitions, hybrid epithelial–mesenchymal (E/M) states and specialised stromal niches, and we distinguish it from oncofoetal reprogramming, an *APC*‐driven programme that CF does not clearly recapitulate. We propose that CF may provide a naturally occurring human context in which chronic epithelial stress sustains EMP‐like pressure, a concept that requires direct validation in human CF intestinal tissue. Finally, we consider how CFTR modulators, microbiome‐directed therapies and redox‐targeted interventions might re‐programme the CF intestinal niche and outline experimental and clinical strategies needed to determine whether early, ecosystem‐level correction can prevent GI cancers in this high‐risk population.

## Graphical abstract

In cystic fibrosis disorder, CFTR dysfunction drives multilayered intestinal niche disruption, including dysbiosis, inflammation, hypoxia, metabolic stress and genotoxic injury, promoting cell‐state plasticity and a pre‐neoplastic ecosystem. CFTR modulators and environmental factors may partially reshape these processes, highlighting opportunities for early intervention in colorectal cancer risk.

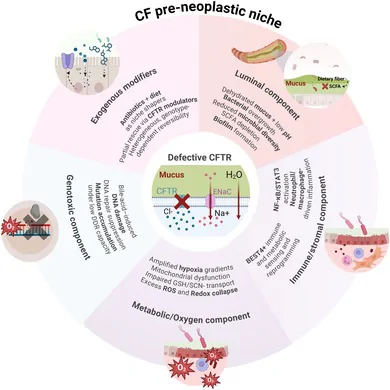

## 1 Introduction: Cystic Fibrosis (CF) Beyond the Lung

CF is a multi‐organ genetic disorder caused by loss‐of‐function variants in the CF transmembrane conductance regulator (*CFTR*) gene, which encodes a multifunctional epithelial ion transporter protein essential for fluid secretion, pH regulation and redox balance across multiple organ systems [1, 2]. Historically defined as a fatal paediatric lung disease, CF is now recognised as a chronic, epithelial disorder affecting the gastrointestinal (GI), pancreatic, hepatobiliary and reproductive systems [2, 3]. Improvement in survival, driven by newborn screening, nutritional optimisation and, more recently, highly effective CFTR modulator therapies, has unmasked a growing burden of non‐pulmonary complications, particularly within the GI tract [3, 4].

CFTR is highly expressed in the developing intestine (colon and small intestinal crypts) and pancreatic epithelia from the first trimester, supporting a prenatal origin of GI abnormalities [5, 6, 7]. Meconium ileus, a neonatal intestinal obstruction caused by primary CFTR‐dependent defects in intestinal epithelial secretion, can be identified in utero on prenatal ultrasound. In parallel, and developing postnatally, impaired CFTR‐dependent chloride (Cl^−^) and bicarbonate (HCO_3_^−^) transport alters the intestinal luminal environment, resulting in dehydrated, hyper‐viscous mucus, acidic luminal pH, impaired enzyme diffusion and dysregulated digestion and microbial colonisation. Consistent with this, approximately 75% of people with CF (pwCF) identified through positive newborn screening have exocrine pancreatic insufficiency at time of diagnosis [3, 8, 9, 10]. Clinically, this manifests as distal intestinal obstruction, meconium ileus, microbial dysbiosis, malabsorption and chronic mucosal inflammation [11, 12]. Over time, these disturbances reshape the intestinal microenvironment into a niche permissive for loss of epithelial integrity, aberrant plasticity and neoplastic transformation.

The intestinal epithelium is one of the tissues with the highest physiological CFTR expression, particularly within the crypt compartment housing the intestinal stem cells (ISCs) that sustain lifelong epithelial renewal [13, 14]. Beyond its canonical Cl^−^/HCO_3_^−^ transport function, CFTR regulates epithelial polarity, barrier integrity, redox homeostasis and stem‐cell behaviour [3, 5, 15, 16]. Loss of CFTR therefore perturbs multiple layers of intestinal biology, extending beyond mucus dehydration to influence carcinogenic susceptibility.

Epidemiological studies consistently demonstrate a striking association between CF and GI malignancies. PwCF exhibit a reported 5–6‐fold increase in risk of CRC [17, 18, 19], with cancers developing at younger ages and often displaying aggressive features; limited data suggest higher short‐term mortality in CF patients hospitalised with CRC; however, robust population‐based survival analyses are lacking [20, 21, 22]. Notably, this risk is further elevated in pwCF post‐lung transplantation [23]. Moreover, heterozygous CFTR variant carriers have been reported in some studies to have a modestly increased risk of CRC and other GI malignancies [19, 24], supporting the concept of CFTR as a tumour suppressor beyond homozygous disease. As a result, CF is now considered a hereditary CRC syndrome, with colonoscopy surveillance recommended from age 40 in pwCF and from age 30 following solid‐organ transplantation [21].

In this review, we propose that CF‐associated CRC risk cannot be explained by mutational burden alone. Rather, CF represents a niche‐centric epithelial disorder in which germline CFTR dysfunction chronically destabilises epithelial identity, disrupts redox and metabolic homeostasis, reshapes microbial and immune interactions, remodels stromal architecture and promotes epithelial–mesenchymal plasticity (EMP). Collectively, we hypothesise that these processes generate a sustained pre‐neoplastic ecosystem in which oncogenic mutations arise within a persistently destabilised epithelial milieu. By integrating evidence from clinical cohorts, animal models, human tissue and patient‐derived organoids, we propose CF as a model of chronic intestinal epithelial instability, while recognising that several components of this framework require direct validation in human CF colonic tissue.

### 1.1 Scope and Search Strategy

This narrative, hypothesis‐generating review was informed by searches of PubMed, Web of Science and Google Scholar through early 2026, supplemented by manual screening of key primary studies, clinical guidelines and major reviews. Search terms combined CF/CFTR with colorectal cancer (CRC), ISCs, BEST4/OTOP2 lineages, EMP or transition, intestinal organoids, microbiome, bile acids, short‐chain fatty acids (SCFAs), inflammation, oxidative stress and CFTR modulators. We prioritised primary human data where available and used murine, airway and organoid studies for mechanistic inference. Formal risk‐of‐bias scoring and quantitative synthesis were not performed. Accordingly, proposed links such as sustained EMP, BEST4^+^ lineage dysfunction and stromal/redox remodelling are presented as a testable framework requiring validation in human CF intestinal tissue.

## 2 The Intestinal Epithelium as a Dynamic, Plastic System

### 2.1 Epithelial Hierarchy and Mesenchymal Niche

The intestinal epithelium is a rapidly self‐renewing tissue organised into crypt‐villus units in the small intestine and crypt‐only structures in the colon, supporting continuous turnover and functional compartmentalisation [25]. Lgr5^+^ ISCs, at crypt base generate transit‐amplifying progenitors that differentiate into absorptive and secretory lineages including enterocytes, goblet cells, Paneth cells, tuft cells, microfold (M) cells and enteroendocrine cells [26]. Renewal is governed by tightly regulated Wnt, Notch, BMP and Hedgehog gradients derived from epithelial and mesenchymal compartments [27, 28].

Recent single‐cell atlases and lineage tracing studies indicate that this hierarchy represents a continuum of states rather than a rigid lineage tree, with injury‐responsive populations and intermediate populations occupying transcriptionally plastic positions between canonical fates [29, 30, 31, 32]. While Wnt/Notch/BMP signalling sustains homeostasis, stress‐associated pathways including TGF‐β, YAP, IFN‐γ and NRG1 drive reprogramming under inflammatory or metabolic perturbation [33, 34]. These transitional states enable regeneration but may also provide a substrate for pathological plasticity in chronic conditions such as CF.

### 2.2 BEST4 ^+^/OTOP2 ^+^ Epithelial Lineages: Ion‐Sensing and Lineage Stability

Single‐cell and spatial transcriptomic studies have identified BEST4^+^/OTOP2^+^ epithelial cells as a distinct human distal colonic lineage enriched for calcium‐activated anion channel (BEST4; Bestrophin‐4) [35, 36] and proton sensing (OTOP2; Otopetrin‐2) [37, 38]. These cells express CFTR alongside guanylin/uroguanylin‐GC‐C signalling components, HCO_3_^−^ transporters and carbonic anhydrases, giving them a distinctive ion‐regulatory signature that positions them as key regulators of luminal pH buffering, fluid secretion and mucosal hydration. Their apical localisation and spatial proximity to the mucus layer further suggest a specialised role in regulating the microenvironment at the epithelial–luminal interface. A recently characterised subset of human small‐intestinal epithelial cells, originally termed CFTR High Expresser (CHE) cells, was subsequently shown to overlap transcriptionally and functionally with BEST4^+^ cells and has been renamed BEST4‐CHE (BCHE) [35, 39, 40, 41]. They co‐express canonical markers of BEST4, CFTR, OTOP2, guanylin/uroguanylin and GC‐C, and demonstrate dynamic apical trafficking of CFTR in response to luminal acidification. This suggests that BEST4‐CHE cells may act as ion‐sensing hubs, integrating Cl^−^/HCO_3_^−^ transport with proton sensing to fine‐tune luminal pH and hydration [35, 40].

Recent functional studies demonstrate BEST4^+^/OTOP2^+^ cells display rapid electrophysiological and transcriptional responses to luminal perturbations and these exhibit cytoskeletal remodelling consistent with active sensing rather than passive buffering [37, 42]. Reduced BEST4 expression in CRC models associates with epithelial–mesenchymal transition (EMT)‐related programmes and increased invasiveness [36, 43], supporting the concept that BEST4^+^ cells contribute to epithelial stability. In CF, where luminal acid–base balance is disrupted, impairment of this lineage may amplify epithelial instability. The mechanistic basis for this stabilising role is becoming clearer, though not without tension in the literature. In CRC cells, BEST4 has been reported to act through a HES4‐TWIST1 axis to suppress EMT. BEST4 expression downregulates the pro‐EMT transcription factor TWIST1, while CRISPR knockout of BEST4 revitalises tumour growth and induces EMT, with low BEST4 expression correlating with advanced stage and worse prognosis [43]. However, other work reports oncogenic activity of BEST4 via PI3K/Akt signalling [36], indicating that BEST4's role may be context‐ and stage‐dependent rather than uniformly protective. Together, these studies support a context‐dependent role for BEST4^+^ cells in epithelial stability, but whether this lineage has a tumour‐suppressive function in the CF intestine remains unresolved. For the CF intestine, where chronic acid–base dysregulation, hypoxia and inflammatory stress may challenge this lineage, the testable implication is that impaired or depleted BEST4^+^/OTOP2^+^ function may lower the threshold for EMP. However, whether BEST4^+^ cells are reduced, dysfunctional or transcriptionally reprogrammed in CF remains unknown and warrants investigation.

### 2.3 Microbiome–Metabolite Interface and Epithelial Buffering

The intestinal mucosa represents a tightly regulated host–microbiome interface. A stratified mucus barrier, enriched in mucins, antimicrobial peptides and secretory IgA, limits microbial contact while permitting metabolic exchange [44]. Commensal microbiota generate SCFAs (butyrate, acetate and propionate) that function as energy substrates and epigenetic signalling molecules regulating mitochondrial metabolism, epithelial differentiation and inflammatory‐tone [45, 46].

Because microbial fermentation alters luminal pH, BEST4^+^/OTOP2^+^ lineages are well positioned to couple metabolite‐driven acidification to epithelial buffering and hydration responses [35, 37, 38, 40, 42]. In vivo vertebrate studies support this axis: in zebrafish, BEST4‐like cells arise within a RANK‐dependent programme shared with tuft/goblet lineages; RANK loss reduces BEST4^+^ cells and enhances inflammatory states, while *Vibrio cholerae* infection expands CFTR^+^ BEST4‐like populations [47].

In CF, where dysbiosis and reduced SCFA availability are reported [48, 49], disruption of this metabolite‐pH sensing‐buffering axis may compound CFTR deficiency, promoting epithelial stress and plasticity.

### 2.4 Stromal Niche and Epithelial–Mesenchymal Crosstalk

The intestinal stem‐cell niche extends well beyond the epithelium to include peri‐cryptal fibroblasts, myofibroblasts, endothelial cells, neurons and immune populations that coordinate epithelial renewal and repair [25, 27]. GLI1‐expressing mesenchymal cells underlie the colonic stem‐cell niche, secrete Wnt ligands required for crypt maintenance; blockade of Wnt secretion from these cells results in stem‐cell loss and crypt collapse, revealing a non‐redundant stromal role in homeostasis [50].

Single‐cell analyses reveal stromal heterogeneity including telocytes, trophocytes, PDGFRA^+^ fibroblasts and inflammatory chemokine‐producing subsets [51, 52, 53]. During tumourigenesis, specialised fibroblast subsets emerge, including RUNX1‐cancer‐associated fibroblasts (CAFs) and pre‐CAFs enriched in early lesions [29]. Early tumour clusters co‐localising with pre‐CAFs are enriched in BEST4^+^ enterocytes, linking epithelial lineage instability to stromal remodelling [29].

A causal role for the niche is supported by the identification of rare pericryptal Ptgs2‐expressing fibroblasts that constitutively produce prostaglandin E_2_ (PGE_2_). Fibroblast‐derived PGE_2_ drives expansion of Sca‐1^+^ reserve‐like stem cells that execute a YAP‐driven regenerative/tumourigenic programme and fibroblast‐specific genetic ablation of Ptgs2 prevented tumour initiation in two independent models of sporadic tumourigenesis [54]. These findings position the mesenchymal niche as an active, paracrine regulator of epithelial plasticity.

In CF, we hypothesise that chronic inflammation, ECM remodelling, hypoxia and dysbiosis may generate a distinctive stromal‐immune environment that interacts with CFTR‐deficient epithelium to bias regeneration towards pro‐plasticity states.

### 2.5 From Epithelial Plasticity to Pre‐Neoplastic Risk

Intestinal epithelial plasticity enables repair following injury. Differentiated lineages, including enterocytes, Paneth and goblet cells, can de‐differentiate and reacquire stem‐like properties under stress [55, 56, 57]. BEST4^+^ cells also display context‐dependent transcriptional shifts under hypoxia or inflammatory cues [36], suggesting participation in regenerative reprogramming.

Persistent or dysregulated plasticity, however, facilitates oncogenic transformation. Single‐cell analyses of colorectal tumourigenesis demonstrate gradual erosion of epithelial identity and emergence of hybrid cell states shaped by immune and stromal signals [29, 30, 31, 32, 58, 59]. These hybrid states exhibit enhanced adaptability and tumour‐initiating capacity [60, 61].

We propose that the same plasticity that enables repair becomes a liability when the epithelium is under chronic stress, as it is in CF. Sustained perturbation of ion transport, mucus hydration, redox balance and microbial composition may bias the CF epithelium towards a persistent regeneration‐associated state rather than allowing return to quiescence, and impaired CFTR‐mediated HCO_3_^−^ secretion and apical polarity may further lower the threshold for aberrant reprogramming. This hypothesis is further explored and developed mechanistically in Sections 4 and 5.

## 3 CFTR as Architect of Intestinal Structure, Redox Balance and Tumour Suppression

### 3.1 Structural Integrity and Epithelial Barrier Regulation

CFTR is expressed throughout the intestinal epithelium [35, 39, 40, 41], enriched within crypt stem and progenitor compartments and localised predominantly to the apical membrane (Figure 1) [3, 14]. Its C‐terminal PDZ‐binding motif anchors CFTR to polarity and scaffolding complexes, including NHERF, ezrin and ZO‐1, integrating it into tight‐junction (TJ) architecture [63, 64, 65]. Disruption of these interactions mislocalises CFTR, alters TJ composition and reduces transepithelial resistance.

**FIGURE 1:**
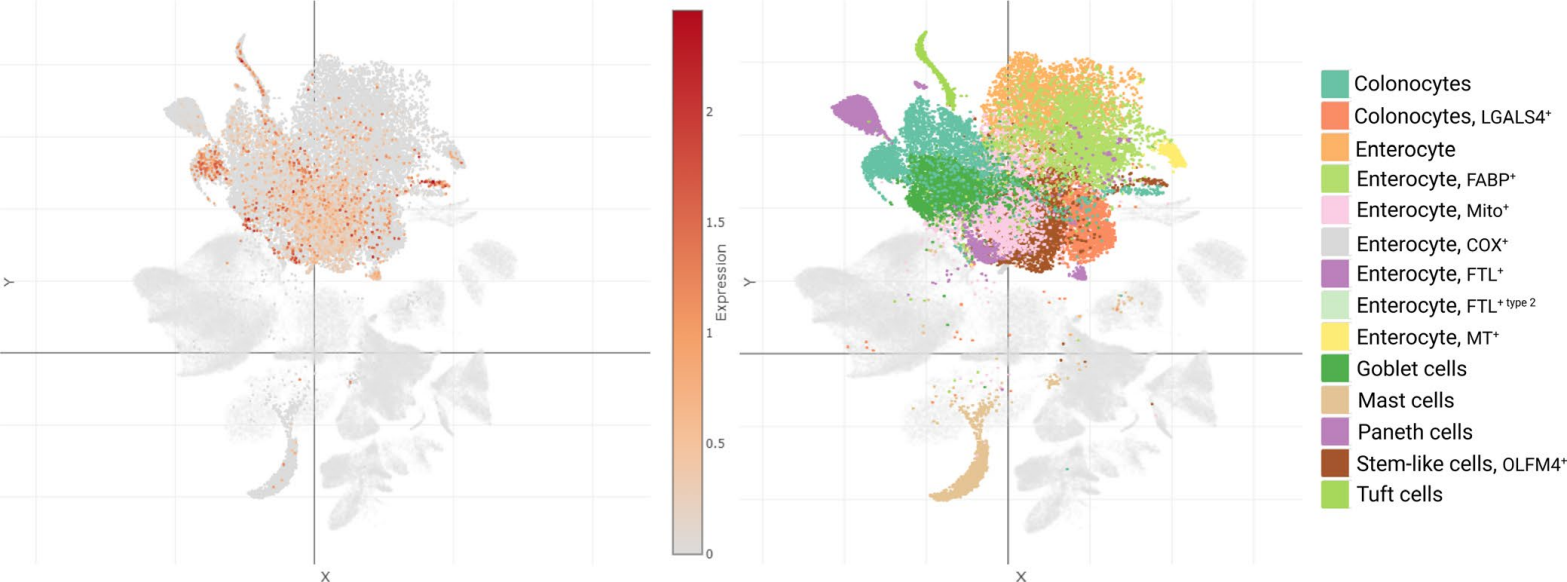
CFTR expression across human epithelial lineages with enrichment in stem and progenitor compartments. CFTR expression in the intestinal epithelium at single‐cell resolution, visualised using publicly available human intestinal single‐cell RNA sequencing data [62], visualised and extracted via the single cell portal interactive platform. Each point represents an individual cell, positioned by uniform manifold approximation and projection (UMAP), a dimensionality‐reduction method that places transcriptionally similar cells close together; the resulting clusters correspond to distinct epithelial cell types. Left panel: *CFTR* transcript abundance per cell overlaid on the UMAP, with colour intensity scaled to normalised expression level (darker/warmer = higher expression; grey/pale = low or absent), illustrating enrichment primarily in stem and enterocyte progenitor populations. Right panel: The same UMAP coloured by annotated epithelial cell identity, with each colour denoting a distinct lineage (e.g., stem/progenitor cells, enterocytes, goblet cells, enteroendocrine cells, BEST4^+^ cells) as defined in the source dataset. Expression values are normalised single‐cell counts as processed in the original study; Cell‐type annotations and clustering derive from the original dataset rather than from independent re‐analysis. No additional statistical testing was performed for this descriptive visualisation.

Across experiential systems, CFTR deficiency leads to disorganisation of cortical F‐actin and microtubules, impaired cell–cell adhesion and increased paracellular permeability [16, 66, 67, 68]. CFTR‐knockout murine intestine exhibits TJs disruption and barrier leak [69]. These structural perturbations resemble early EMT‐like features suggesting that CFTR loss lowers the threshold for epithelial identity destabilisation under niche stress.

### 3.2 Ion Transport, pH Buffering and Intrinsic Redox Control

CFTR mediates Cl^−^/HCO_3_^−^ secretion, establishing osmotic gradient that hydrates mucus and regulates luminal viscosity [70]. It also exerts inhibitory control over epithelial sodium channels (ENaC), limiting excessive sodium and water reabsorption [71]. In the intestine, impaired HCO_3_^−^ secretion lowers luminal pH, disrupts mucin expansion and reduces digestive enzyme diffusion, promoting obstruction and bacterial overgrowth [3].

Beyond ion transport, CFTR supports apical redox defence, including transport of glutathione (GSH) and thiocyanate (SCN^−^). Reduced antioxidant capacity is documented in CF airway and intestinal surfaces, increasing susceptibility to reactive oxygen species (ROS) mediated damage [15, 72, 73].

Importantly, oxidative stress may arise as a cell‐autonomous consequence of CFTR loss. CFTR knockdown in intestinal epithelial cells induces mitochondrial dysfunction, disturbed fatty‐acid metabolism and downregulation of DNA repair pathways independent of inflammation [74]. These findings position CFTR at the intersection of epithelial ion regulation and intrinsic redox stability.

### 3.3 Stem‐Cell Regulation and Tumour Suppression

CFTR influences ISC dynamics. CFTR‐deficient murine intestines demonstrate hyperactivated Wnt/β‐catenin signalling, increased ISC proliferation and impaired differentiation [75, 76, 77]. While a distinct stable stem‐cell population is not observed, these models consistently show altered stable stem‐cell state regulation and expansion of proliferative crypt compartments. In *APC*‐wild‐type *CFTR*‐knockout mice, spontaneous intestinal tumours arise at a significant frequency by 1 year of age, supporting a tumour‐suppressive role for CFTR independent of canonical *APC* mutations [65].

These murine findings should, however, be interpreted with appropriate caution. Mouse and human intestine differ in CFTR expression patterns, crypt architecture, intestinal physiology and several experimental models rely on genetically engineered or chemically induced backgrounds [69, 75, 76, 77], including AOM‐DSS, that may not fully recapitulate the slow, spontaneous trajectory of human CF‐associated tumourigenesis. Rodent models also lack a human‐specific gut microbiome, limiting inference about microbiota‐dependent mechanisms central to the niche model proposed here. Finally, much of the available evidence derives from F508del‐homozygous or *CFTR*‐null animals, and whether these findings extend to residual‐function variants or other CFTR genotypes remains unresolved. Thus, murine data establish that CFTR loss can drive intestinal tumourigenesis in principle, but the quantitative translation to human CF risk requires confirmation in primary human tissue, patient‐derived organoids and genotype‐stratified cohorts.

Emerging evidence from organoid models highlight its role in epithelial plasticity. CFTR activation promotes maturation of preterm human intestinal epithelium, driving differentiation towards adult enterocyte programmes including BEST4^+^ lineages [78]. CFTR inhibition maintains an immature epithelial state and impairs lineage progression. This suggests that CFTR may constrain epithelial plasticity by stabilising transitions from progenitor to terminally differentiated states.

In sporadic CRC, CFTR expression is frequently reduced, often through promoter hypermethylation [79, 80]. CFTR polymorphisms are enriched in young‐onset CRC and serrated polyposis syndrome [79, 80]. Low CFTR expression correlates with poorer disease‐free survival and increased metastatic potential, and CFTR‐depleted CRC cell lines exhibit enhanced migration and clonogenicity [81]. These data support the involvement of CFTR in context‐dependent maintenance of epithelial state fidelity and thereby potentially modulating tumour progression. More emerging evidence is explored in Sections 4 and 5.

### 3.4 Genome Instability and Mutational Susceptibility

While CFTR is not a classical DNA repair gene, its loss destabilises genome maintenance through redox and metabolic dysregulations. Oxidative stress promotes mutagenic lesions including 8‐oxo‐deoxyguanosine and replication stress, which accumulate when antioxidant defences are reduced [15, 74]. CFTR‐deficient IECs exhibit mitochondrial dysfunction and downregulation of DNA repair enzymes, even in sterile conditions [74], indicating that genome instability may precede inflammatory insult.

The mutual trajectory of CF‐associated intestinal neoplasia remains incompletely defined. Available evidence does not support a uniform, FAP‐like pathway dominated by early *APC* loss, nor does it fully mirror the IBD‐associated carcinogenesis. Instead, CFTR dysfunction appears to act upstream of canonical oncogenic mutations by lowering the threshold for mutation fixation, within a chronically destabilised crypt niche. In this framework, *APC* loss may represent a selected event within an already plastic and redox‐compromised epithelium rather than an obligate initiating mutation.

We therefore propose that CFTR dysfunction primes the intestinal epithelium for oncogenic evolution through niche‐mediated genome instability, coupling cell‐autonomous oxidative stress, impaired repair resilience and sustained epithelial plasticity.

## 4 Intestinal Plasticity, EMP and Cell‐State Reprogramming in Colorectal Tumourigenesis

Intestinal tumourigenesis is increasingly viewed as progressive epithelial state instability rather than a single stepwise genetic transition. In the canonical *APC*‐driven pathway, early oncogenic stress induces de‐differentiation and reactivation of foetal or injury‐associated transcriptional programmes, a process termed oncofoetal reprogramming [82]. This preserves epithelial identity while shifting cells towards regenerative, stem‐like state that supports adenoma initiation [82, 83].

As tumours evolve, persistent inflammatory, metabolic and mechanical stress broadens plasticity. Advanced lesions display lineage infidelity, hybrid differentiation states and progressive EMP, enabling invasion and stress tolerance. In sporadic, *APC*‐driven CRC these programmes appear temporally stratified, with oncofoetal reprogramming most prominent at tumour initiation and sustained EMP underpinning progression and heterogeneity. As discussed below (Section 4.3), current CF data do not establish that an oncofoetal programme is engaged in the CF intestine. We, therefore, invoke oncofoetal reprogramming here as a comparator drawn from sporadic CRC rather than as a demonstrated feature of CF.

### 4.1 Regenerative Plasticity and Niche‐Driven Invasion

Regenerative plasticity provides a permissive cellular substrate for early tumour reprogramming. Under severe injury, differentiated IECs can reacquire stem‐like features [55, 56, 57]. Under conditions of *APC* loss or chronic inflammation, Paneth cells and ISCs can re‐enter proliferative pools and fuel adenoma formation [31, 58].

Importantly, canonical mutations alone do not confer invasion. Colorectal organoids harbouring *APC*, *Kras* and *Trp53* mutations remain non‐invasive until exposed to TGF‐β, which induces epithelial plasticity and lineage reprogramming [84]. These data underscore that niche‐derived signals may be crucial for transition from in situ neoplasia to invasive carcinoma.

### 4.2 EMT, Hybrid States and Dynamic Stemness

Classical EMT involves loss of polarity and acquisition of mesenchymal traits [85, 86]. In CRC, however, complete EMT is uncommon. Instead, tumour cells occupy hybrid epithelial/mesenchymal (E/M) states, with a dynamic EMP continuum [87, 88]. These hybrid states enhance adaptability, stress tolerance and metastatic competence [61, 87, 89].

Experimental evidence indicates that fully mesenchymal states may compromise tumour‐initiating capacity, whereas hybrid EMP states preserve the balance between self‐renewal and motility required for dissemination [87, 88, 89]. Lineage‐tracing studies further show that disseminating colorectal tumour cells are often Lgr5^−^ but can reacquire Lgr5 expression upon colonisation, reconstituting hierarchical tumour structure [90]. Thus, stemness and plasticity, in this context, can be dynamically regulated rather than remaining fixed properties.

In contrast, oncofoetal reprogramming promotes regenerative metabolic and chromatin states that enhance proliferation and stress tolerance but do not inherently confer invasion [82, 83]. Together, these observations suggest that epithelial plasticity in sporadic CRC operates through distinct but complementary programmes (oncofoetal‐like reprogramming supporting regenerative expansion and hybrid EMP states supporting dissemination) with stemness gained and lost dynamically. Therefore, the exact components of these cellular programmes, and whether they are engaged sequentially or in parallel during tumour progression will need to be resolved in the context of CF.

### 4.3 CF as a Proposed Model of Sustained EMP Pressure

Within CF, many EMP‐promoting pressures are amplified. CF epithelia are exposed to chronic oxidative stress, thick mucus, dysbiosis, altered SCFA availability and recurrent inflammation—all converging on HIF‐1α, NF‐κB, STAT3, YAP/TAZ and TGF‐β/SMAD pathways that implicate plasticity and neoplastic transformation [65, 91]. These signalling networks converge on key transcriptional regulators of EMP including TWIST1 and YAP1, which act as downstream integrators of inflammatory, metabolic and mechanical stress [67, 91]. At present, these molecular changes are most consistent with stress‐induced epithelial plasticity and EMP rather than definitive activation of an oncofoetal transcriptional programme. In contrast, *APC* loss is a central driver of oncofoetal reprogramming in intestinal tumourigenesis, but its interaction with CFTR dysfunction has not been directly examined [83, 92]. CFTR‐deficient airway models demonstrate EMT‐like changes with reduced E‐cadherin, increased vimentin and cytoskeletal disorganisation [16, 67, 93]. Furthermore, our previous findings suggest that the EMT phenotype is dependent on the specific CFTR variant and functional CFTR ion transport, as loss of CFTR ion transport, despite the physical presence of (non‐functional) CFTR at the plasma membrane, is enough to trigger partial EMT and is associated with upregulation of TWIST1 and YAP1 in CF airway epithelial cells [93].

Together, these findings motivate a CF‐specific hypothesis aligned with contemporary CRC frameworks: CF‐associated tumourigenesis may be driven primarily by chronic, CFTR‐centred microenvironment pressure that destabilises epithelial identity and sustains hybrid EMP states, rather than enforcing a fixed lineage switch. In this model, classical driver mutations arise on a stress‐primed epithelial landscape rather than being the sole initiators. The proliferative driver in this framework remains to be defined. Sustained EMP and stress adaptation are proposed to render the epithelium permissive to transformation, but whether proliferative expansion is supplied primarily by conventional oncogenic lesions acquired on this destabilised background, by niche‐derived pro‐proliferative signalling such as fibroblast PGE_2_‐YAP activity [54], or by loss of CFTR‐intrinsic proliferative restraint cannot be resolved from current data. Distinguishing these possibilities and determining whether they act independently or in combination will require direct experimental testing in CF‐relevant models.

### 4.4 CF Relative to Other CRC‐Associated Conditions

Several other conditions are associated with increased CRC risk via distinct but overlapping mechanisms, including inflammatory bowel disease (IBD), primary sclerosing cholangitis‐associated colitis, coeliac disease, familial adenomatous polyposis (FAP) and Lynch syndrome [94, 95, 96] (Table 1). These comparators help distinguish CF from both classical mutation‐first hereditary CRC syndromes and inflammation‐first colitis‐associated cancer pathways.

**TABLE 1:** Positioning CF within cancer‐prone intestinal diseases: Mechanistic drivers, niche features and surveillance logic.

Condition	Initiating lesion	Dominant risk architecture	CRC risk/age pattern	Tumour location/molecular phenotype	Inflammation contribution	Microbiome/luminal chemistry contribution	Epithelial state/plasticity angle	Typical pathway framing	Surveillance logic (high‐level)	Key references (this manuscript)
CF	Germline CFTR dysfunction (epithelial transport + stability node)	Niche + state pressure with mutational accumulation over time; hereditary CRC syndrome framing does not fully explain risk	Reported several‐fold increased CRC risk, with earlier onset than the general population; risk further increased post‐transplant	Predominantly right‐sided; CF‐associated driver landscape and molecular phenotype remain incompletely characterised	Often present early; linked to barrier/mucus dysfunction, microbe contact and stromal remodelling	Dehydrated/acidic mucus; dysbiosis; SCFA loss; bile‐acid alterations	Chronic stress shapes regenerative programmes and EMP propensity; CFTR has acts context‐dependent tumour suppressor functions	Niche‐centric predisposition with ecosystem disruption	CF‐specific colonoscopy recommendations including earlier screening post‐transplant	[3, 12, 21, 50, 51, 52, 53, 54, 57, 65, 74, 91, 97, 98, 99, 100, 101, 102, 103, 104, 105, 106]
IBD (UC/Crohn's colitis)	Chronic mucosal immune dysregulation	Inflammation‐driven field effect + genomic instability	Increased risk associated with disease duration, extent and inflammatory burden	Often associated with inflamed colonic segments; molecular evolution differs from sporadic CRC	Central driver (cytokines, oxidative stress)	Dysbiosis and barrier disruption are important modifiers	Repeated injury/repair cycles promote de‐differentiation, stromal remodelling and EMT/EMP programmes	Colitis‐associated cancer (CAC) pathway	Risk‐stratified surveillance based on duration/extent and other factors	[94, 95, 107]
Lynch syndrome	Germline mismatch repair (MMR) defect → microsatellite instability	Mutation‐driven (accelerated mutational burden)	High CRC risk with earlier onset than sporadic CRC	Predominantly right‐sided; MSI‐high phenotype	Not required (can be present but not primary)	Secondary/modifier	State programmes arise downstream of high mutation rate; tumour evolution shaped by immune context	Hypermutated pathway framing	Guideline‐driven surveillance due to high genetic risk	[96]
FAP (*APC* pathway)	Germline *APC* dysfunction	Mutation‐driven initiation with large polyp burden	Very high CRC risk without intervention; onset typically young	APC‐driven adenoma–carcinoma sequence	Not primary	Secondary/modifier	Plasticity/EMP likely shapes progression, but initiation is strongly genetically determined	Adenoma‐carcinoma sequence	Intensive surveillance/prophylaxis logic due to polyp burden	[95, 99]
Serrated polyposis/young‐onset CRC gene enrichment	Heterogeneous germline predisposition variants in cancer‐associated genes	Mixed (genetic predisposition with distinct lesion biology)	Variable risk and age of onset depending on phenotype	Often right‐sided serrated lesions; CIMP/BRAF‐associated subsets	Variable	Potential modifier; varies with phenotype	Plasticity/state programmes may differ; context‐dependent	Serrated pathway framing	Surveillance based on polyp burden/pattern and risk features	[58, 59]

Abbreviations: CAC, colitis‐associated cancer; CF, cystic fibrosis; CRC, colorectal cancer; FAP, familial adenomatous polyposis; IBD, inflammatory bowel disease; MMR, mismatch repair.

In Lynch syndrome and FAP, germline defects in mismatch‐repair genes or *APC*, respectively, accelerate accumulation of oncogenic mutations with comparatively less dependence on chronic inflammation. However, unlike FAP or Lynch syndrome, key CRC‐driving mutations in CF have not been systematically defined. To date, no studies have comprehensively characterised the genetic landscape of CF‐associated polyps, limiting firm conclusions about the molecular sequence of tumour initiation. Thus, CF cannot yet be assigned to a canonical mutation‐first pathway analogous to Lynch syndrome or FAP.

Because CF and IBD share chronic mucosal inflammation, dysbiosis and barrier dysfunction, their CRC pathways may appear similar; however, the initiating lesion and causal direction differ fundamentally. In IBD‐associated colitis, inflammation is the primary driver: an immune‐led, relapsing–remitting process produces mucosal field defects, early TP53 mutation and inflammation‐associated EMT, with dysbiosis and epithelial injury largely downstream of immune activation [107]. In CF, by contrast, the initiating lesion is a germline epithelial ion‐transport defect that alters luminal pH, mucus hydration, bile‐acid handling and epithelial polarity from early life. Dysbiosis and inflammation in CF are therefore at least partly downstream of a primary epithelial–luminal abnormality, rather than being the initiating event.

This distinction has three implications. First, the CF epithelial defect is lifelong and epithelial‐intrinsic, whereas IBD inflammation is typically acquired and immune‐initiated [65, 76]. Second, CF intestinal inflammation is chronic but not classically relapsing–remitting in the manner of ulcerative colitis or Crohn's colitis, and may therefore generate a different type of epithelial stress field from the intense cytokine‐driven field cancerisation described in colitis‐associated cancer [107]. Third, and most distinctively, CFTR appears to have context‐dependent tumour‐suppressive functions in the intestinal epithelium, including effects on Wnt restraint, DNA‐repair and redox regulation. Its loss may therefore combine a hereditary‐syndrome‐like epithelial predisposition with an IBD‐like inflammatory and microbial microenvironment.

Available data suggest that CF‐associated tumourigenesis does not follow a uniform, mutation‐first trajectory and alterations in pathways commonly deregulated in CRC, including KRAS, TGF‐β, EGFR and TP53, remain poorly explored in the CF context and warrant systematic investigation [108]. We propose that CF is best understood as a distinct niche‐centric CRC‐risk state, in which multi‐layered epithelial ecosystem failure and chronic EMP pressure may create a permissive pre‐neoplastic field rather than acting through either a purely mutation‐first or purely inflammation‐first pathway.

### 4.5 Disentangling Primary CFTR‐Driven Effects From Secondary Confounders

A central interpretive challenge is that CF is a complex multi‐system disease, and several of the intestinal features we attribute to CFTR dysfunction (dysbiosis, SCFA depletion, altered bile‐acid pools, chronic mucosal inflammation) are also plausibly shaped by secondary factors associated with the CF phenotype. Exocrine pancreatic insufficiency alters nutrient delivery, fat absorption and bile‐acid handling [109]; recurrent broad‐spectrum antibiotic exposure for pulmonary infection profoundly remodels the gut microbiota [110]; high‐energy, high‐fat CF diets and nutritional supplementation modify luminal substrate availability [111] and CF‐related diabetes and hepatobiliary disease introduce systemic metabolic perturbations [112]. Each of these can independently influence the luminal, metabolic and inflammatory layers of the niche we describe and disaggregating their contributions from cell‐intrinsic CFTR loss is currently difficult in human cohorts. We therefore caution against attributing the full pre‐neoplastic phenotype to CFTR dysfunction alone.

The strongest evidence that CFTR loss exerts primary, cell‐intrinsic effects comes from reductionist systems that hold these confounders constant: in cultured intestinal epithelial cells, CFTR deletion is sufficient to induce mitochondrial dysfunction, disrupted lipid/fatty‐acid homeostasis and downregulated DNA‐repair pathways in the absence of microbes or an immune compartment [74]. Conversely, characteristic CF intestinal phenotypes are not all epithelial‐autonomous. In the *CFTR*‐knockout intestine, goblet cell hyperplasia requires non‐epithelial input rather than arising cell‐intrinsically [113], indicating that some features attributed to CFTR loss are in fact niche‐ and microbe‐driven, an explicit example of a secondary rather than primary effect.

In this framework, CFTR dysfunction is treated as the initiating epithelial lesion, whereas pancreatic insufficiency, antibiotic exposure, diet, CF‐related diabetes and hepatobiliary disease are secondary modifiers that may amplify or redirect the intestinal niche response. The primary‐versus‐secondary distinction is experimentally tractable. Genotype‐matched organoid systems [114, 115, 116], microbe‐controlled animal models [117, 118] and human analyses that account for pancreatic status, antibiotic exposure and CF‐related comorbidities could each help apportion the contributions of cell‐autonomous CFTR loss versus secondary factors. This distinction is not merely a caveat, as it bears on which features of the niche are likely correctable by CFTR restoration and which would require microbiome‐, diet‐ or inflammation‐directed approaches.

## 5 Building a CF‐Specific Pre‐Neoplastic Niche

CFTR dysfunction does not act in isolation. Available evidence suggests that luminal, immune, metabolic/oxygen and genotoxic disturbances may reinforce one another over time, supporting the concept of a CF‐specific ‘pre‐neoplastic intestinal niche’ (Table 2 and Figure 2).

**TABLE 2:** Evidence matrix for a CF‐specific pre‐neoplastic intestinal niche: Components, mechanisms and tractable readouts.

Niche layer (section)	Primary CF‐associated perturbation	Key evidence base (human/animal/epithelial models)	Mechanistic link to plasticity/pre‐neoplasia	Modifiable levers	Suggested assays/biomarkers (translatable)	Key gaps/testable predictions
Luminal component	Dehydrated, acidic mucus; altered mucin expansion; microbial dysbiosis; biofilm propensity	CF intestine physiology [3, 12]; altered gut microbiota in adults with CF [11]; CFTR/CRC synthesis [91]; mucosa‐attached microbiome changes [100]; host gene regulation + microbiome interactions [98] and dysbiosis overview [119]	Acidic, stagnant mucus + dysbiosis increases epithelial–microbe contact and chronic regenerative signalling pressure [3, 44, 98, 100, 120]	Diet/fibre and fermentable substrates [31, 40]; antibiotics [82]; CFTR modulators [98, 99, 100, 102, 103, 119, 121]	Stool: SCFAs [45, 46, 49], inflammatory indices [103, 113, 121]; microbiome profiling [11, 98, 100, 122, 123, 124, 125, 126, 127]	Does SCFA restoration track with reduced epithelial stress/EMP programmes in mucosa? Are biofilm‐associated features relatively non‐reversible despite ETI?
Epithelial barrier/polarity	Tight junction disruption; permeability; loss of epithelial stability scaffolds	GI localisation of CFTR [14]; CFTR PDZ/polarisation and tight junction regulation [63, 64]; CFTR tumour suppressor gene in intestine [65]; CFTR‐junction/cytoskeleton synthesis [16] and CFTR KO permeability/junction phenotypes [69, 77]	Barrier leak promotes immune activation; junction/cytoskeletal remodelling lowers threshold for EMP‐like transitions [16, 64, 65, 69]	CFTR modulators [113, 122, 123, 124, 125, 126, 127, 128, 129]; anti‐inflammatory strategies [103, 121]	Biopsy: junction proteins (e.g., ZO‐1 pathways) [64]; epithelial permeability surrogates; organoids/monolayers TEER; junction gene signatures [69]	Which barrier defects normalise with ETI vs. persist due to stromal/immune memory or long‐standing niche remodelling?
Immune/stromal component	Chronic mucosal inflammation (often early, even without overt injury)	Intestinal inflammation in CF [103]; chronic inflammation in morphologically normal CF intestine [121]; inflammation‐CRC framework [107]; CFTR/CRC review [91]	NF‐κB/cytokine signalling as well as stromal signalling can bias de‐differentiation and regenerative programmes that facilitate tumour initiation [50, 51, 52, 53, 54, 57, 58, 107]	CFTR modulators [113, 122, 123, 124, 125, 126, 127]; targeted anti‐inflammatory approaches (clinical context)	Stool markers (e.g., calprotectin) [103, 113, 121]; mucosal cytokine panels; immune cell profiling (single‐cell/spatial where feasible)	Does reduction in stool inflammatory markers with ETI reliably track reduced epithelial stress/EMP/DDR signatures in mucosa?
Metabolic/oxygen/redox component	Hypoxia microdomains; mitochondrial stress; oxidative stress; impaired antioxidant capacity	Hypoxia impact on IEC function [106]; systemic glutathione deficiency in CF [73]; oxidant stress suppresses CFTR [72]; CFTR deletion causes mitochondrial dysfunction/lipid disruption [74]; CFTR multifunctionality [15, 24]	Hypoxia + ROS support stress‐adapted hybrid states (EMP) and increase damage burden; reduced redox buffering lowers resilience to stress [74, 106, 130]	Redox‐targeted approaches; diet/metabolites [45, 46, 48]; CFTR modulators [113, 122, 123, 124, 125, 126, 127, 128, 129]	Oxidative stress and mitochondrial markers in biopsies/models; organoid hypoxia‐challenge phenotypes; integrated stool metabolomics	Are distinct ‘EMP‐prone’ epithelial states enriched in CF crypts under hypoxia/ROS and partially reversible with ETI?
Genotoxic component	Bile acid shift + ROS/hypoxia → DNA damage plus reduced repair resilience	Bile acids as endogenous etiologic agents [97]; DCA induces mitochondrial ROS and NF‐κB [131]; bile acid malabsorption in CF [101]; toxic bile acids in pancreatic‐insufficient CF [104]; stool bile acid profiling [132]; DDR overview [133]; hypoxia effects on metastasis gene expression [134]; genomic instability hallmark [124] and bile‐acid induced micronuclei/mitotic perturbations [125]	Convergent ‘high damage/low repair’ pressure increases mutational opportunity and supports malignant evolution on a stress‐primed epithelium	Diet composition; bile acid‐binding/conditioning strategies (clinical consideration); microbiome‐directed approaches; modulators indirectly	Stool bile acid panels [101, 104, 132]; mucosal DDR activation markers; γH2AX/53BP1 in models; oxidative DNA lesion readouts	Do bile acid profiles + hypoxia/redox markers stratify advanced lesion risk independent of inflammation?
Exogenous modifiers	Antibiotics, diet and modulators variably reshape upstream layers	Diet and gut–lung axis in CF [48]; ivacaftor effects on gut microbiota/inflammation [113]; extended ETI effects [122]; pilot modulator microbiome study [126]; ETI clinical efficacy anchor [123]; longitudinal ETI microbiome profiling [127] and mixed diversity findings after ETI [128, 129]	Modulators may partially re‐normalise luminal chemistry and inflammatory tone; heterogeneity likely reflects ecological ‘locking in’ and exposure history	ETI and earlier modulators [113, 122, 123, 124, 125, 126, 127, 128, 129]; dietary strategies [48] and antibiotic stewardship	Longitudinal stool microbiome/metabolites/inflammation paired with functional epithelial assays (e.g., organoids)	Key unknown: does early modulator initiation prevent niche establishment and reduce advanced lesions/CRC incidence over time?

Abbreviations: DDR, DNA damage response; EMP, epithelial–mesenchymal plasticity; ETI, elexacaftor/tezacaftor/ivacaftor; pwCF, people with CF; SCFA, short‐chain fatty acids.

**FIGURE 2:**
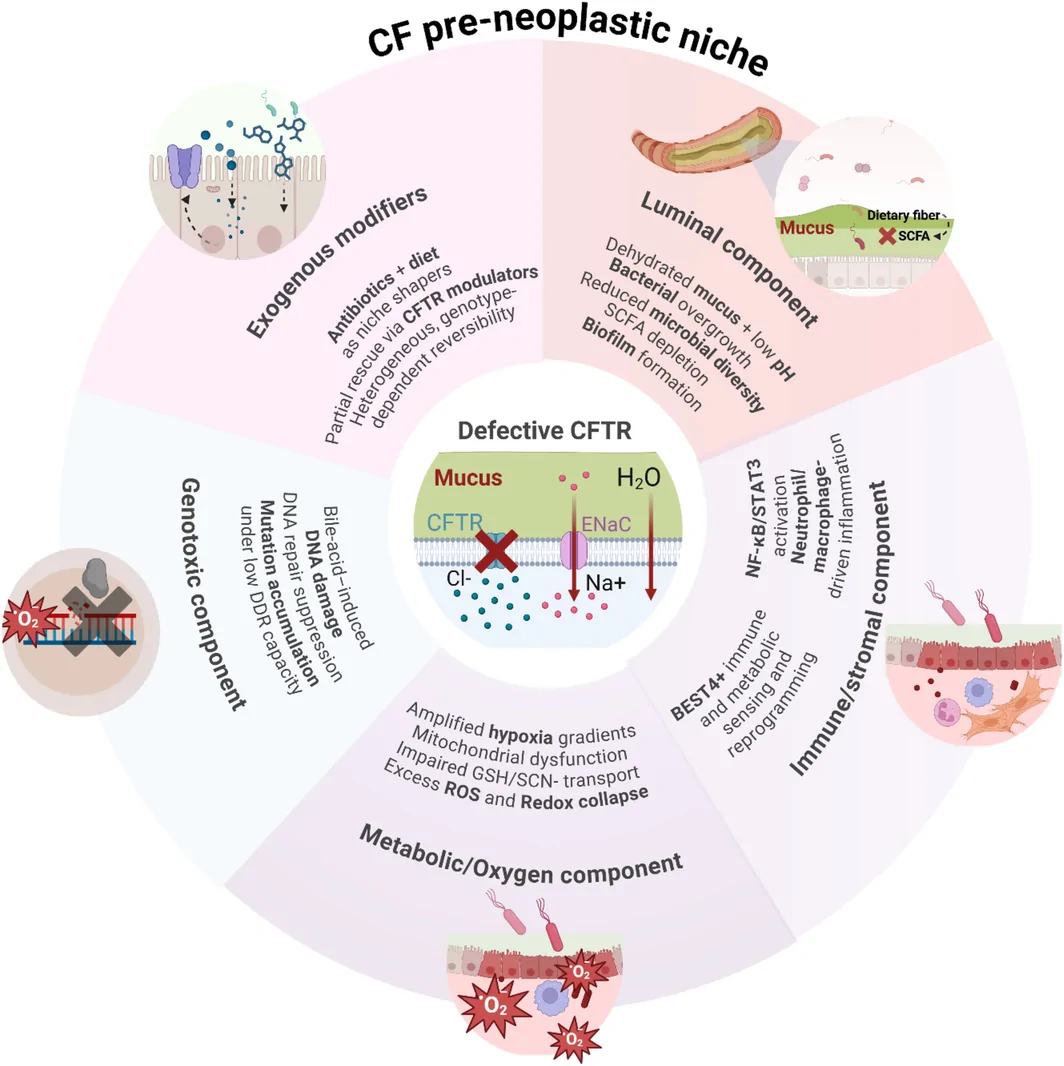
CFTR dysfunction and the intestinal niche: a multi‐layer model of pre‐neoplastic risk. CFTR dysfunction is proposed to drive a multilayered intestinal ecosystem disruption that can develop into a pre‐neoplastic niche. The model distinguishes primary CFTR‐intrinsic epithelial effects from secondary CF‐associated modifiers, including pancreatic insufficiency, antibiotic exposure, diet, CF‐related diabetes and hepatobiliary disease, which may amplify or redirect the intestinal niche response. (1) Luminal component: Impaired Cl‐/HCO3‐ secretion contributes to dehydrated mucus, acidic luminal pH and impaired mucin expansion, associating with dysbiosis (loss of SCFA‐producing taxa, expansion of pathobionts) and biofilm formation that can perpetuate barrier injury and chronic regenerative pressure. (2) Immune/stromal component: Barrier leak and dysbiosis can promote chronic innate immune activation (often neutrophil‐skewed), with NF‐κB‐linked signalling capable of biasing epithelial de‐differentiation and β‐catenin/Wnt activation. BEST4^+^/CFTR‐high epithelial lineages may integrate luminal, microbial and metabolic cues and could contribute to epithelial buffering responses and cell‐state instability under stress. (3) Metabolic/oxygen component: Mucus stasis, microbial overgrowth and inflammation can deepen local hypoxia, promoting mitochondrial stress, impaired antioxidant defences and ROS accumulation that may shift epithelial cells towards stress‐adapted states with increased EMP propensity. (4) Genotoxic component: Elevated bile acids, ROS and hypoxia may increase DNA damage while also reducing repair capacity, creating a high‐injury/low‐repair environment that could increase mutational burden over time. (5) Exogenous modifiers: Antibiotics, diet and CFTR modulators can reshape these layers to varying degrees, either exacerbating dysbiosis/metabolic stress or partially restoring luminal hydration, inflammatory tone and epithelial stability—highlighting opportunities for early ecosystem‐level intervention.

### 5.1 Luminal Component: Mucus Stasis, Biofilm, Dysbiosis and SCFA Loss

In the healthy colon, a dense inner mucus layer is largely bacteria‐free, while an outer layer houses diverse commensals that metabolise dietary substrates into SCFAs and vitamins [25, 44]. CFTR loss impairs Cl^−^/HCO_3_^−^ secretion, reducing mucus hydration, altering mucin expansion and lowering pH, which hinders enzyme diffusion. This reshapes microbiome–mucus interactions that maintain gut–microbiome homeostasis [3].

CFTR‐knockout mice display ~40‐fold increased bacterial load and reduced species diversity in the small intestine, with expansion of Escherichia/Enterobacteriaceae and loss of secondary bile‐acid metabolising genera such as Ruminococcus and Enterorhabdus [91, 102, 119]. Similar patterns of dysbiosis are reported in pwCF, with depletion of SCFA‐producing taxa (e.g., Bifidobacterium, Roseburia, Faecalibacterium) and enrichment of taxa associated with CRC risk (e.g., Actinobacteria and pathogenic Clostridium clusters) [100, 119]. Dayama et al. integrated mucosal RNA‐seq and 16S rRNA profiling to show that CF colonic mucosa has > 1500 differentially expressed genes enriched for CRC‐related pathways (p53, mTOR, metastasis) and reduced butyrate‐producing bacteria, with increased taxa associated with CRC development [91, 98]. Loss of butyrate, a key fuel for colonocytes and epigenetic regulator of gene expression, likely contributes directly to pro‐tumourigenic reprogramming [91].

A further consequence of reduced microbial diversity and altered mucus architecture is bacterial biofilm formation, which protects bacteria from antibiotics and immune clearance and impairs nutrient absorption [119]. Biofilm‐forming CF‐associated organisms (such as *Pseudomonas aeruginosa* , *Staphylococcus aureus* and *Escherichia coli* ) can degrade mucins, release proteases and toxins, impair antibiotic penetration and disrupt TJs, perpetuating a cycle of dysbiosis, barrier injury and inflammation [91, 119]. Over time, this constitutes a stable luminal platform for chronic injury and regenerative pressure.

### 5.2 Immune Component: Chronic Inflammation and Stromal Remodelling

The CF intestine exhibits evidence of chronic immune activation from early life, characterised by elevated IL‐8, IL‐1β, immunoglobulins, neutrophil elastase and eosinophil cationic protein in stool and mucosal samples, consistent with sustained neutrophilic and eosinophilic activity [103, 121]. Notably, inflammation is frequently observed in the absence of overt villus injury, implicating epithelial barrier leak, aberrant immune sensing of luminal microbes and dietary antigens as primary drivers rather than secondary responses to tissue damage [121].

Comparative gene set enrichment analyses of CFTR‐knockout and Muc2‐knockout intestines reveal overlapping inflammatory and stress‐response signatures, mechanistically linking CFTR dysfunction, mucus abnormalities and dysbiosis to a shared pro‐tumourigenic immune landscape [65]. However, CFTR‐deficient intestinal organoids maintained under sterile conditions do not display strong intrinsic inflammatory programmes, indicating that inflammation in CF is largely niche‐driven and emerges from epithelial–microbial–stromal interactions rather than being strictly cell autonomous [130].

NF‐κB activation can enhance Wnt signalling and induce de‐differentiation of non‐stem cells into tumour‐initiating cells, accelerating adenoma formation in mouse models [58]. Persistent exposure to TNF‐α, IL‐6 and IL‐11, together with barrier dysfunction, plausibly drives a similar regenerative and de‐differentiation bias in the CF intestine. Than et al. proposed that dysbiosis‐driven inflammation synergises with CFTR loss to activate β‐catenin signalling, mirroring the azoxymethane‐dextran sodium sulphate (AOM‐DSS) model of colitis‐associated tumour promotion [65].

Within this inflammatory milieu, stromal remodelling is increasingly recognised as a critical mediator of epithelial fate [135]. Under homeostatic conditions., subepithelial fibroblasts are essential regulators of ISC behaviour, providing spatially organised Wnt ligands, R‐spondins, BMP antagonists and ECM cues that balance renewal and differentiation along the crypt axis [50, 53, 136]. In chronic inflammatory contexts, however, fibroblasts undergo phenotypic activation, acquiring altered secretome and ECM‐producing programmes that disrupt crypt architecture and homeostatic niche signalling [135, 137]. Activated fibroblasts secrete cytokines, chemokines, proteases and matrix components that increase tissue stiffness, distort morphogen gradients and promote epithelial dedifferentiation and regenerative bias, thereby lowering the threshold for tumour‐initiating states, particularly in genetically susceptible epithelium [135, 138].

Functional studies also reveal that TGF‐β‐activated fibroblasts promote tumour initiation and expand the frequency of tumour‐initiating cells, and that this effect is shared across all poor‐prognosis CRC subtypes, driven by a common TGF‐β‐induced programme in the stroma [139]. Importantly, inhibition of TGF‐β signalling to disrupt tumour‐stroma cross‐talk halts disease progression in patient‐derived organoids and xenografts, highlighting stromal TGF‐β signalling as a key mediator of aggressive CRC biology [139]. CRC subtypes display distinct immune landscapes: CMS1 tumours that exhibit strong immune infiltration and more responsive to checkpoint blockade, whereas CMS4 tumours that are mesenchymal/stroma‐rich, TGF‐β‐driven, with poorer prognosis [140]. These frameworks suggest that chronic inflammation and stromal remodelling in CF may not merely accompany epithelial instability but actively structure a niche that biases regeneration, immune tolerance and early neoplastic risk [140]. Notably, these stromal mechanisms characterised in sporadic CRC or IBD [135, 139] have not yet been resolved in CF specifically. Whether CF fibroblasts acquire the activated, niche‐disrupting phenotype described in colitis remains an untested but addressable question. This represents a specific gap that single‐cell and spatial profiling of the CF colon could close.

Finally, CRC risk is amplified after solid‐organ transplantation. People with CF who undergo lung transplantation carry a markedly higher CRC risk than non‐transplant pwCF, variously estimated at 20–30 times the general‐population rate [21], and a substantially elevated risk even relative to other transplant recipients [141]. This excess is thought to reflect the inherent CF predisposition compounded by intense, sustained immunosuppression [141]. This suggests that immunosuppression‐driven loss of tumour immune surveillance, particularly in the stromal niche, may also play an integral role in shaping cancer risk in CF.

### 5.3 Metabolic/Oxygen Component: Hypoxia, Mitochondrial Stress and Redox Failure

The intestinal epithelium operates under steep oxygen gradients: ~7%–10% O_2_ in muscularis, ~6% in the submucosa and ~2% at the crypt interface—‘physioxia’ [106]. Chronic inflammation, impaired perfusion, mucus stasis and microbial overgrowth can push local oxygen below this range, creating hypoxic microdomains. In CF, thick mucus and dysbiosis are predicted to exaggerate hypoxia and oxidative stress [91, 106].

CF tissues exhibit reduced GSH:GSSG ratios, diminished SCN^−^ secretion and elevated ROS [15, 72, 73]. Kleme et al. demonstrated that CFTR loss in IECs is sufficient to trigger oxidative stress through mitochondrial dysfunction and impaired DNA repair [74], positioning redox failure as both a cell‐autonomous vulnerability and a niche‐amplified stress.

### 5.4 Genotoxic Component: Bile Acids Combined With ROS/Hypoxia Leads to DNA Damage and Repair Stress

At the niche level, CF creates chronic genotoxic pressure through convergent luminal and microenvironment insults. Pancreatic insufficiency, bile acid malabsorption and fat‐enriched diets generate a luminal milieu with increased concentrations of primary and secondary bile acids [3, 12]. Microbial conversion of primary bile acids into secondary species such as deoxycholic acid (DCA) and lithocholic acid (LCA) produces detergents that can damage epithelial membranes, generate ROS and induce DNA strand breaks [97, 131]. These can activate NF‐κB and EGFR/MAPK pathways, promote COX‐2 expression and induce chromosomal instability and aneuploidy in colonic epithelium [97, 105].

Although detailed bile‐acid profiling in CF colons remains limited, several studies report altered bile‐acid composition and increased faecal bile acids in CF and in exocrine pancreatic insufficiency more broadly [101, 104, 132]. In parallel, ROS generated through inflammation and impaired antioxidant export produces mutagenic lesions such as 8‐oxo‐dG, single‐ and double‐strand breaks and replication stress [133]. Hypoxia further suppresses homologous recombination and mismatch repair [134], compounding damage accumulation. Crucially, CFTR‐deficient IECs show reduced expression of multiple DNA damage response genes (e.g., FANCD2, RAD51, XRCC1) [74], suggesting that CF crypts may face a dual burden of high damage load plus limited repair capacity or a persistent repair‐injury state.

### 5.5 Modifiers and Partial Reversibility

A defining feature of the CF pre‐neoplastic niche is that many drivers are modifiable. Recurrent broad‐spectrum antibiotic exposure perturbs commensal communities and may perpetuate dysbiosis [91]. Diet composition influences SCFA production and bile‐acid pools, directly acting on luminal and metabolic layers.

CFTR modulators uniquely target the imitating defect. Clinical studies of lumacaftor/ivacaftor and ETI report improvements in GI symptoms, reductions in stool inflammatory markers (including faecal calprotectin in many cohorts) and partial restoration of SCFA‐producing taxa [113, 122, 126]. Effects vary with genotype, pancreatic status, antibiotic exposure and age at CFTR modulator initiation, and many individuals lack effective modulator options. Thus, CFTR restoration may function as an ecosystem‐level intervention, but its capacity to stabilise epithelial cell state and reduce long‐term neoplastic risk remains unresolved.

## 6 CFTR Modulators as Ecosystem and Risk Modifier

The advent of highly effective CFTR modulators, particularly the triple combination Elexacaftor/Tezacaftor/Ivacaftor (ETI), has transformed CF care by improving CFTR folding, trafficking and gating, and producing clinically meaningful effects across multiple organ systems [123]. In an intestinal context, this creates a testable premise: if CFTR loss helps establish a pre‐neoplastic ecosystem, then CFTR rescue may partially ‘re‐normalise’ upstream niche conditions including pH, mucus hydration, microbial structure, inflammatory tone, thereby reducing chronic pressure towards epithelial reprogramming. Hoverer, only approximately 5–6 years of real‐world ETI exposure are currently available, an observation window far shorter than the 10–15‐year latency over which the adenoma‐carcinoma sequence typically unfolds [142, 143]. Direct evidence linking modulators to altered CRC incidence is therefore not yet available, and current interference relies on intermediate niche endpoints rather than cancer outcomes.

### 6.1 Microbiome and Luminal Ecology

Across multiple cohorts, ETI has been associated with shifts towards a more balanced microbial profile, including abundance of SCFA producing bacteria such as Faecalibacterium and Roseburia and reduced representation of potential pathobionts, suggesting partial correction of dysbiosis [113, 122, 127]. However, intestinal microbial diversity has been reported to remain unchanged in some cohorts [128, 129], highlighting that symbiotic microbial function may be constrained by long‐standing ecological remodelling, antibiotic history and early‐life imprinting [128, 129]. These mixed findings suggest that modulator‐driven luminal improvement may be real but partial, and whether it translates into durable epithelial stabilisation, rather than transient compositional change, remains unresolved.

### 6.2 Inflammation and Epithelial Stress

Stool inflammatory markers (including calprotectin and M2‐pyruvate kinase) decrease in many, but not all, patients after modulator therapy [113]. This variability is consistent with a layered model in which some components of the niche may be readily modifiable with improved hydration and pH, while others (biofilms, stromal/immune memory, established metabolic rewiring) may persist despite CFTR rescue.

Whether CFTR modulators initiated in adulthood can reverse an intestinal niche established through decades of CFTR dysfunction remains unknown; correction of hydration and inflammation may be achievable, whereas accumulated DNA damage, stromal remodelling and stable epithelial cell‐state changes may be only partially reversible.

### 6.3 Epithelial Plasticity

Short‐term organoid and in vitro epithelial studies suggest that CFTR functional restoration can stabilise epithelial identity under stress conditions, including hypoxia‐induced EMP. In in vitro epithelial models, ETI reduces vimentin expression and EMT‐like morphology, paralleling observations in CF airway epithelial cells [67, 93]. Conceptually, from an EMP perspective, CFTR modulators may act not only as ion‐transport correctors but also as partial anti‐plasticity agents. However, this remains a hypothesis requiring direct validation in CF intestine. There are currently no in vivo intestinal data demonstrating that modulators reverse Wnt hyperactivation, oxidative DNA damage, BEST4^+^ lineage dysfunction or established crypt plasticity. The available evidence is short‐term and largely based on organoid, airway or in vitro systems, with emerging proteomic evidence of cell‐state change. These findings provide proof‐of‐principle that niche correction is biologically plausible, but they do not yet establish cancer‐risk reduction.

### 6.4 Timing and Completeness of Rescue

Major uncertainties remain. CFTR rescue is incomplete in many tissues; some CFTR mutations respond sub‐optimally and modulator access is not universal. Importantly, timing is also likely to be critical. Modulators initiated early in life, before a pre‐neoplastic niche consolidates, might prevent or attenuate niche establishment, whereas later initiation may only partially reverse an ecosystem that has already undergone microbial, inflammatory, stromal and epithelial remodelling.

### 6.5 CFTR Heterozygote Carriers and Gene‐Dosage Effects

The question of CRC risk in CFTR heterozygote carriers is clinically important but remains unresolved. Some epidemiological studies report modestly elevated CRC risk even in heterozygous CFTR variant carriers [24, 91, 144], implying that reduced CFTR dosage alone may be sufficient to destabilise epithelial identity and biologically relevant. However, these associations remain inconsistent and may be influenced by ascertainment bias, population stratification and incomplete adjustment for shared exposures. This means it cannot yet be treated as a settled effect, a heterogeneity that mirrors the variable microbiome responses after modulator therapy [128, 129]. In addition, modulators rarely restore CFTR function to wild‐type levels, and clinical improvement in pulmonary or nutritional outcomes does not necessarily imply complete correction of the residual epithelial deficit relevant to long‐term cancer risk. Resolving the carrier‐risk question and defining the threshold of CFTR restoration required for epithelial stabilisation will therefore be important, although current evidence does not support specific CRC surveillance recommendations for carriers.

### 6.6 Sex and CRC Risk

Current evidence suggests that, despite advances in modulator efficacy, the female sex seems to be an independent predictor of worse overall outcomes in CF [145] (called the CF ‘gender gap’). No study has yet examined whether CRC incidence, age of onset or tumour location in CF might be sex‐dependent, particularly given the interaction of sex hormones with both CF disease severity and colonic epithelial biology [146].

### 6.7 Implications for Surveillance and Cancer Risk

No published colonoscopy cohort yet has the duration or statistical power to detect a change in adenoma or carcinoma incidence attributable to modulators, and existing CF‐CRC screening recommendations were formulated largely in the pre‐ETI period [21, 147]. Preliminary surveillance data from the NICE‐CF study reported broadly similar rates of non‐advanced precancerous polyps and advanced neoplasia among individuals receiving CFTR modulators and those not receiving modulators. However, the available follow‐up remains short relative to the natural history of colorectal carcinogenesis, and these findings should not be interpreted as evidence that ETI has no effect on future CRC risk [148]. Due to this limited follow‐up, long‐term effects on cancer risk remain formally unquantified since the advent of modulators [147]. The net direction of effect is also unclear. What is clear is that CFTR modulators extend survival in the CF population [17]. So, even if modulators protect the intestinal functioning at a cellular level, it will be imperative to delineate its effect on niche remodelling and how that may shape CRC risk, especially in the ETI‐treated CF population.

We, therefore, view current organoid and microbiome data as proof‐of‐principle that niche remodelling is possible, but not yet as definitive evidence of cancer risk reduction. Prospective colonoscopy‐linked cohorts combined with longitudinal multi‐omic profiling will be essential to determine whether modulator‐era CF shifts the intestinal trajectory away from chronic EMP pressure and neoplastic risk.

## 7 Future Directions and Translational Roadmap

The next phase is to convert the ecosystem model into causal human evidence. High‐resolution mapping of the CF intestine using single‐cell and spatial multi‐omics should define how CFTR loss reshapes epithelial cell states, stromal programmes and immune architecture across age and treatment exposure. The central question is not only which cell types change, but whether CF establishes persistent hybrid or injury‐biased states that lower the threshold for EMP.

Mechanistic testing can be advanced using patient‐derived organoids and organoid‐microbe platforms to dissect how pH, SCFA depletion, bile acids, hypoxia and inflammatory cytokines interact with CFTR genotype to drive plasticity. These systems allow identification of which niche inputs are necessary, sufficient and reversible.

Translation will depend on upstream risk stratification. If CF is a niche‐driven cancer‐prone condition, surveillance strategies should incorporate layered biomarkers (microbiome/SCFA profiles, bile acids, stool inflammatory indices, epithelial stress markers) alongside conventional colonoscopy. The modulator era offers a natural experiment to determine whether restoring CFTR function rewinds the niche and stabilises epithelial identity in vivo. These principles are beginning to be tested clinically, though no trial uses CRC or its precursors as an endpoint. The PROMISE study, the first large prospective programme to characterise the multi‐system effects of ETI, has now reported GI findings: 6 months of ETI was associated with measurable shifts in faecal microbial composition and reductions in markers of intestinal inflammation, alongside improvements in microbiome diversity [128]. The PROMISE‐GI analysis found that patient‐reported GI symptoms, though improved on some measures, changed only modestly from an already low baseline [149]. Complementary paediatric cohorts report increased intestinal microbiome diversity and reduced microbial antibiotic‐resistance and acid‐tolerance gene abundance post‐ETI—consistent with a less acidic, more CFTR‐corrected luminal environment [127]. In parallel, microbiome‐directed approaches have entered controlled testing: randomised trials of probiotics (e.g., *Lactobacillus* GG) report reduced faecal calprotectin and partial microbiota restoration in children with CF [150], and the PEARL‐CF trial was designed explicitly to test whether early‐life microbiome modulation can act before intestinal microbiota maturation [151], directly probing the ‘therapeutic window’ implied by our niche model. The critical unmet need is to extend these intermediate endpoints towards neoplastic ones. Future trials should incorporate longitudinal epithelial, microbial and inflammatory biomarkers, and ultimately with adenoma/carcinoma incidence, to determine whether ecosystem‐level correction modifies the trajectory towards CRC rather than only improving short‐term GI physiology.

## 8 Conclusion

CFTR loss is associated with disruption of epithelial structure, luminal ecology, immune tone and redox balance that collectively may reshape the intestinal niche towards chronic regenerative pressure and heightened epithelial plasticity. Evidence from murine models, human tissues and CF organoids supports a testable model in which CF‐associated colorectal risk may emerge not solely from accelerated mutation accumulation, but from sustained niche‐driven destabilisation of epithelial identity.

We propose that CF is best understood as a human model of chronic, ion‐transport‐centred niche failure, in which persistent microenvironmental stress lowers the threshold for malignant transformation. Whether CFTR restoration can recalibrate this ecosystem and modify long‐term cancer risk remains an urgent and testable question in the modulator era.

## Author Contributions

S.A.W. conceived the review and outlined the scope. B.U. and S.A.W. wrote the first draft. I.P., M.A., C.Y.O., T.J. and S.A.W. provided critical intellectual input and edited/revised the manuscript. All authors reviewed and approved the final version.

## Funding

This work was supported in part by an National Health and Medical Research Council grant (NHMRC_APP1188987) and Sydney Children Hospital Network Foundation and Luminesce Alliance Research.

## Conflicts of Interest

The authors declare no conflicts of interest.

## Data Availability

The data that support the findings of this study are available from the corresponding author upon reasonable request.
